# Supplementary material for: Activation of an Effective Immune Response after Yellow Fever Vaccination Is Associated with the Genetic Background and Early Response of IFN-γ and CLEC5A
Source: Viruses. 2021 Jan 12;13(1):96. doi: 10.3390/v13010096 (PMC7828179; doi:10.3390/v13010096)
Supplement: Supplementary file 1 [file viruses-13-00096-s001.zip › Supplementary Figure 1.docx]

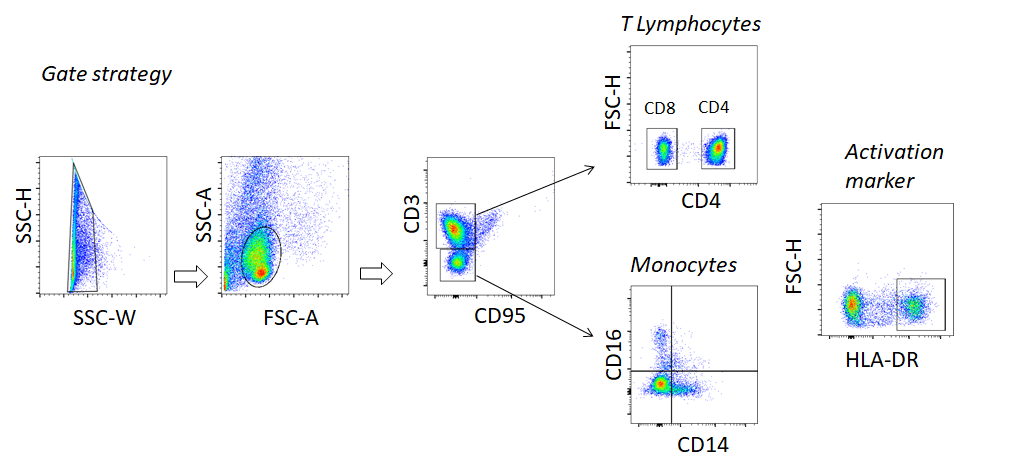


**Supplementary Figure S1.** Gating strategy for activated T lymphocytes and monocytes by flow cytometry. Representation of plots used to select and analyze live CD4^+^ (CD3^+^CD95^-^CD4^+^) and CD8^+^(CD3^+^CD95^-^CD4^-^) T cells, and live monocytes (CD3^-^CD95^-^CD14^+^ and CD3^-^CD95^-^CD14^+^CD16^+^), expressing the activated marker, HLA-DR, after antigen stimulation *in vitro* to evaluate the immune response after YF vaccination.
