# Supplementary material for: Activation of an Effective Immune Response after Yellow Fever Vaccination Is Associated with the Genetic Background and Early Response of IFN-γ and CLEC5A
Source: Viruses. 2021 Jan 12;13(1):96. doi: 10.3390/v13010096 (PMC7828179; doi:10.3390/v13010096)
Supplement: Supplementary file 1 [file viruses-13-00096-s001.zip › Supplementary Figure 2.docx]

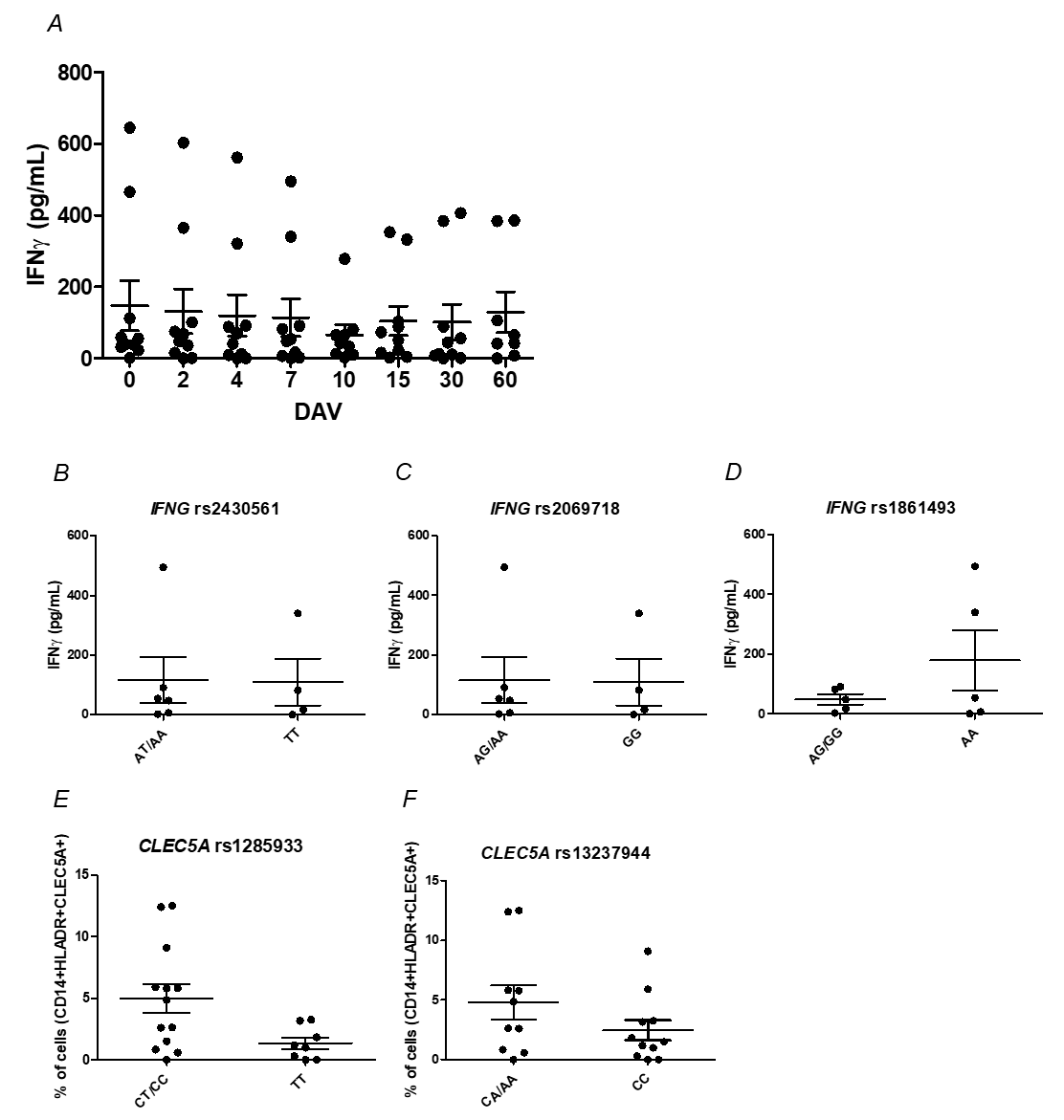


**Supplementary Figure S2.** CLEC5A and IFN-γ protein levels according SNPs analyzed. (A) Circulating levels of IFN-γ early times after vaccination (n=10). (B-D) Circulating levels of IFN-γ at 7 DAV was stratified according to *IFNG* (B) rs2430561, (C) rs2069718, and (D) rs1861493 genotypes. (E-F) Frequency of *ex vivo* activated monocytes expressing CLEC5A at 5 DAV, according to *CLEC5A* (E) rs1285933 and (F) rs13237944 genotypes (n=21). Each dot corresponds to one individual analyzed, with median and standard error of groups. Statistical analysis was determined using Mann-Whitey test. P value is represented as * *p* < 0.05, ** *p* < 0.01. DAV- Days After Vaccination.
