# Supplementary material for: Activation of an Effective Immune Response after Yellow Fever Vaccination Is Associated with the Genetic Background and Early Response of IFN-γ and CLEC5A
Source: Viruses. 2021 Jan 12;13(1):96. doi: 10.3390/v13010096 (PMC7828179; doi:10.3390/v13010096)
Supplement: Supplementary file 1 [file viruses-13-00096-s001.zip › Supplementary Table 1_revised.docx]

**Supplementary Table S1.** Descriptions of genes, main immunological roles and primer pairs

| **Symbol** | **Name** | **Immunological role** | **Primer sequence** |
| --- | --- | --- | --- |
| *CLEC5A* | C-Type Lectin Domain Containing 5A | Myeloid immune receptor; proinflammatory response [31,34,37] | 3'-TCATTTGGCATTCTTCTCACAG-5' |
|  |  |  | 5'-GGCAATGTTACCAATCAGAATCA-3' |
| *DAP12* | DNAX-Activation Protein 12 | CLEC5A accessory protein; proinflammatory response [31,34,37] | 3'-TCGGTCTCAGTGATACGCT-5' |
|  |  |  | 5'-GTAAGTGGTCTCCGTCCTGTC-3' |
| *STAT1* | Signal Transducer And Activator Of Transcription 1 | Transcription of antiviral genes in response to interferons [54] | 3'-ATGGGCTTCATCAGCAAGGA-5' |
|  |  |  | 5'-AACCGCATGGAAGTCAGGTT-3' |
| *STAT2* | Signal Transducer And Activator Of Transcription 2 | Transcription of antiviral genes in response to interferons [54] | 3'-AAACCTCATCCACGGTGTTC-5' |
|  |  |  | 5'-CCAGATTTGCCCTGTGATCT-3' |
| *IRF7* | Interferon Regulatory Factor 7 | Transcription of antiviral genes in response to interferons [54] | 3'-CCTGTGGTGGTGGGACAG-5' |
|  |  |  | 5'-TGATGCTGCGGGATAACTC-3' |
| *IRF9* | Interferon Regulatory Factor 9 | Transcription of antiviral genes in response to interferons [54] | 3'-AGGCTCTACACCAGGGACAG-5' |
|  |  |  | 5'-TCCAGCCATACTCCACAGAA-3' |
| *OAS1* | 2'-5'-Oligoadenylate Synthetase 1 | Interferon-induced antiviral enzyme [55] | 3'-ATCGTCGGTCTCATCGTCTG-5' |
|  |  |  | 5'-GGCTGAATTACCCATGCTTTA-3' |
| *RNASEL* | Ribonuclease L | Interferon-induced antiviral enzyme [56] | 3'-CGGATGAACTTTAGCAGATCAC-5' |
|  |  |  | 5'-ACCTGGGCCTTCTGAACATT-3' |
| *IL6* | Interleukin 6 | Proinflammatory cytokine [57] | 3'-GAAAATCATCACTGGTCTTTTGG-5' |
|  |  |  | 5'-GCATCTAGATTCTTTGCCTTTTT-3' |
| *IL12* | Interleukin 12 | Proinflammatory cytokine [57] | 3'-GTGGAGGTCAGCTGGGAGTA-5' |
|  |  |  | 5'-TTTCTTTTCTCTCTTGCTCTTGC-3' |
| *CXCL10* | C-X-C Motif Chemokine Ligand 10 | Chemokine; leukocyte recruitment [58] | 3'-TTCTTGATGGCCTTCGATTC-5' |
|  |  |  | 5'-CCAATTTTGTCCACGTGTTG-3' |
| *AIM2* | Absent In Melanoma 2 | Interferon-induced gene; Inflammation [59] | 3'-GCTTAGACCAGTTGGCTTGA-5' |
|  |  |  | 5'-CATCTGGAGTTCATAGCACCATAA-3' |
| *IFI16* | Interferon Gamma Inducible Protein 16 | Interferon-induced gene; Inflammation [59] | 3'-TTTCTTGTTTTTCCTGGTCTTGA-5' |
|  |  |  | 5'-TTGAATTGGCACCGAAAAGT-3' |
| *IFNGR1* | Interferon Gamma Receptor 1 | Subunit 1 of IFN𝛾 receptor [18] | 3'-AAAGGAGGTGGGGGCTTTTATTACG-5' |
|  |  |  | 5'-TGGTCTGTGAAGAGCCGTTGTCTCC-3' |
| *IFNG* | Interferon Gamma | Antiviral activity; Induction of adaptive response [18] | 3'-ATTGCTTTGCGTTGGACATT-5' |
|  |  |  | 5'-TGACCAGAGCATCCAAAAGA-3' |
| *RPL13* | Ribosomal Protein L13 | Constitutive expression | 3'-GTACTTCCAGCCAACCTCG-5' |
|  |  |  | 5'-GACAAGAAAAAGCGGATGGT-3' |
| *GAPDH* | Glyceraldehyde-3-Phosphate Dehydrogenase | Constitutive expression | 3'-CAACAGCCTCAAGATCATCAGCAA-5' |
|  |  |  | 5'-AGTGATGGCATGGACTGTGGTCAT-3' |
