# Supplementary material for: Activation of an Effective Immune Response after Yellow Fever Vaccination Is Associated with the Genetic Background and Early Response of IFN-γ and CLEC5A
Source: Viruses. 2021 Jan 12;13(1):96. doi: 10.3390/v13010096 (PMC7828179; doi:10.3390/v13010096)
Supplement: Supplementary file 1 [file viruses-13-00096-s001.zip › Supplementary Table 2.docx]

**Supplementary Table S2.** Selected SNPs descriptions

| **Chr** | **SNP ID** | **(GRCh37)** | **A1** | **A2** | **Allele frequency (A2)** | **Gene** | **Functional Consequence** |
| --- | --- | --- | --- | --- | --- | --- | --- |
| 7 | rs13237944 | 141639215 | C | A | 0.25619 | *CLEC5A* | Intronic |
| 7 | rs1285933 | 141627149 | G | A | 0.458866 | *CLEC5A* | 3’ UTR |
| 12 | rs2430561 | 68552522 | T | A | 0.280152 | *IFNG* | Intronic |
| 12 | rs2069718 | 68550162 | A | G | 0.383187 | *IFNG* | Intronic |
| 12 | rs1861493 | 68551196 | A | G | 0.209465 | *IFNG* | Intronic |
